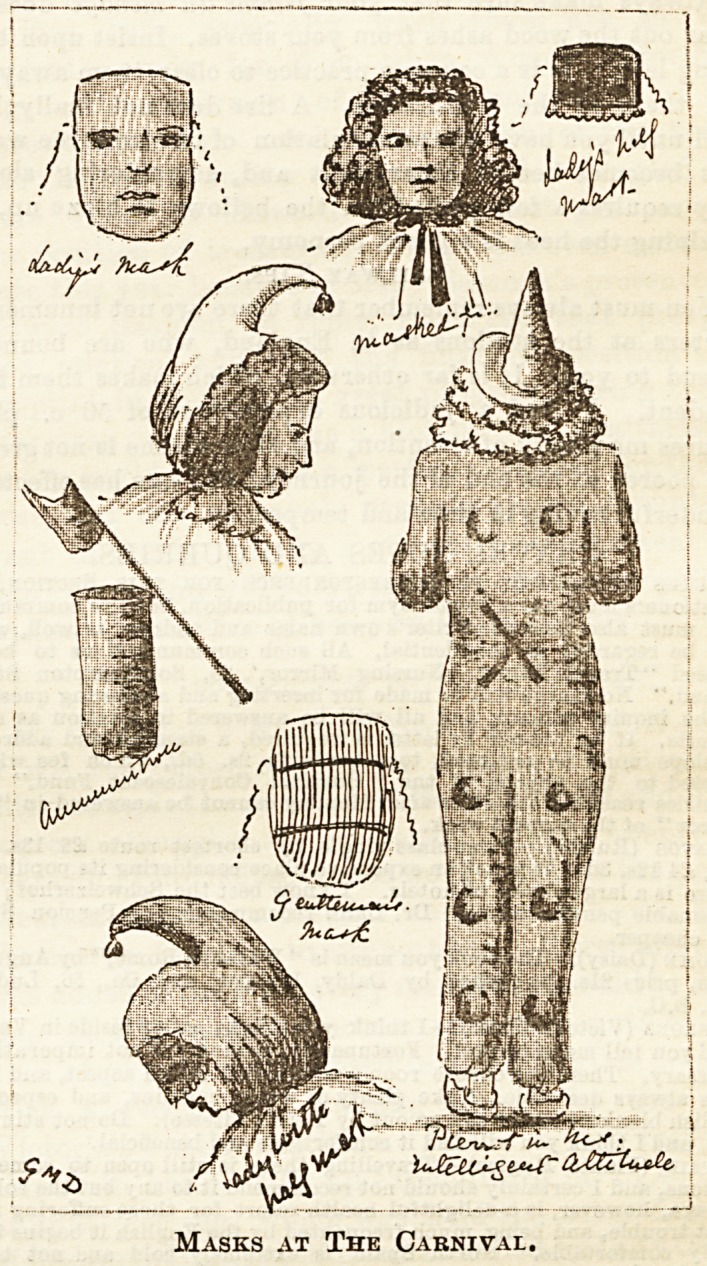# "The Hospital" Nursing Mirror

**Published:** 1899-02-11

**Authors:** 


### "The Hospital" Nursing Mirror.

Being the Nursing Section of "The Hospital."

[Contributions for this Section of "The Hospital" should be addressed to the Editor, The Hospital, 28 & 29, Southampton Street, Strand, London, W.C., and should have the word "Nursing" plainly written in left-hand top corner of the envelope.]

### News from the Nursing World.

### PRINCESS CHRISTIAN AT CAMBRIDGE.

On the 2nd inst. Princess Christian visited Cambridge and opened a bazaar at the Town Hall in aid of the fund to provide a convalescent home for the Addenbrooke's Hospital. She was met at the railway station by the Lord Lieutenant of the County and Miss Peckover, the High Sheriff and Mrs. F. Crisp, the Mayor, Mayoress, and Miss Kett, who presented a bouquet. After luncheon at Trinity College the Princess proceeded to the Guildhall, attended by a guard of honour of the University Rifle Volunteers, where she was welcomed by a large assembly. The Lord Lieutenant said that the visit of the Princess was a great honour and encouragement to them. The Queen had ever shown herself deeply interested in all institutions provided for the relief of sickness and suffering, and the Royal Family constantly followed her example. For 20 years Addenbrooke's Hospital had been a training school for nurses, and many hundreds of probationers passed through its wards. Although the hospital needed financial assistance, which it was difficult to obtain in a country district suffering from agricultural depression, it had nevertheless been thought right to provide a convalescent home at Hunstanton, because it had been found that the patients made a better recovery by the seaside. An anonymous friend had promised to give them the house, and the financial equipment of the home was secured for three years. There was already one convalescent home connected with the hospital, but it was insufficient for their needs. In the first place, it was generally full, and surgical cases were not admitted. Princess Christian then declared the bazaar open. Her Royal Highness left the Guildhall about half-past four amid hearty cheering from a large crowd collected in the market-place, and returned to town by train.

### THE NURSES' GUERDON.

It is just about twenty years since nursing was recognised as a skilled art. Many of the women who then took up nursing as their mission in life, fired by the enthusiasm and example of Florence Nightingale, have now reached the limits of their powers of work; age has fallen upon them, and an exceeding bitter cry is heard from many quarters, "How and where can we spend our latter days in comfort?" "What guerdon is there for us in the sabbath of our days?" The answer is a pitiless one. Unless out of the daily remuneration received during the time of their activity a competency has been saved there are but two alternatives—charity or the workhouse. Because this is so, we would that each nurse, actual and prospective, should strip bare of sentiment the ugly facts of a nurse's career; that she should with open eyes and with calm judgment view them in the light of cold common sense, and make her arrangements accordingly. These are the things she must take into consideration. The span of her working time is short—about 20 years only after her training is finished—the nature of her work entails a strain that incapacitates her continuing it when her vitality fails; and, even if her strength permitted it, it is with difficulty that she can then obtain employment. How many nurses there are to-day who dare not be older than 35, and to whom grey hair would mean destitution. What we wish to draw attention to is this: that the nominal pay which a nurse receives is only received for a comparatively few years, and that out of it she ought to subtract enough to provide for her age. The pay attached to her work is not large. The earnings of the most lucky are from £75 to £100 a year, but from £30 to £60 may be taken as a fair average. Out of this amount about half must be saved if anything like an adequate provision is to be made. Can, then, the remaining half be made to provide for dress, recreation, and the necessary expenses involved in the waits between cases and in holidays? Are you yourself willing to make this sum do it? For, should the age limit of work be reached, without having due provision made for it, the wolf of destitution is at the door.

### MAL-ORGANISATION.

To our English notions constitutional representation is desirable in government, but there is plenty of evidence that an absolute monarch is not always a bad ruler, and that for constitutional government to succeed there must be unity of aim and method. It were not unprofitable to ask, Is there always unity of aim and method in the government of our hospitals and infirmaries? In this light, we have just been reading the rules for the government of the North Staffordshire Infirmary and Eye Hospital. It is practically managed by a weekly committee, and this weekly committee is appointed every month by the general committee. Obviously there can be very little permanent unity of purpose in a committee the members of which may be changed from month to month. Following on to the management of the nursing and domestic affairs it becomes apparent that the lady superintendent has no authority over a perfectly independent housekeeper, and yet at the same time it is decreed that "she shall be charged with the general oversight of the domestic working of the institution, and in case of observing any irregularity or shortcomings in it, she shall call the attention of the housekeeper to the same." How profitable and pleasant such a task must be! Let it also be noticed that, whilst the lady superintendent and the housekeeper are appointed by the general committee, the superintendent of night nurses is appointed by the weekly committee! Therefore, so far as these rules are carried out, the infirmary is managed by a group of perfectly independent officials, and it is the duty of each to keep an eye on the others—in other words, to spy and interfere. Yet no one has any authority to enforce a "suggestion" or to administer a reprimand. It is easy to see that the position of the lady superintendent could be made untenable. Her night superintendent, whilst nominally under her control, is appointed by the weekly committee, to whom she can carry complaints if she is dissatisfied, whilst the housekeeper could quietly nullify all her authority and ignore her direct requests. Nevertheless, in spite of these anomalies, she is charged with the "domestic working of the institution" (the rules do not say to whom she is responsible, presumably to the general committee), whilst she is responsible to the honorary medical staff for all that concerns the patients—diet-sheets, food, &c.!

### THE AFTER CARE ASSOCIATION.

FOR many reasons the "After Care Association" for those who are discharged cured from asylums merits the attention of those engaged in nursing. In the first place it is a necessary and useful society, filling in, as it does, the horrible chasm that lies between the asylum doors and the patient's return to freedom; its aid can be most valuable to those who nurse, especially amongst the poor; it is capable of many developments which should widen the field for nursing services; and lastly it has what may be an important bearing on nurses who are laying down their active work and who possess cottage homes. The association is desirous of adding to its list of homes suitable for the reception of recovered mental patients until their perfect health be re-established, and who could be more suitable for this work than kindly elderly nurses? It must come as a boon to those who desire some occupation in their retirement, and even if the pay is not large a few shillings to the good every week makes the difference between just enough and plenty. Bonâ-fide nurses should apply to the secretary of the association, Church House, Dean's Yard, Westminster. The secretary inspects the homes periodically, and a lady visitor is appointed by the council, so that the burden of responsibility is shared by persons experienced in dealing with such cases; whilst, of course, it limits the homes where such persons are sent to the really suitable ones. It must also be remembered that the patients are already cured, and only require judicious sympathy and care.

### COMBINED CERTIFICATES.

THE cost of advertising is really becoming a heavy burden to the ratepayers of the unfortunate Poor-law districts which possess no training schools. The Strabane Board (Belfast) have lately spent no less than £14 in advertising for a second nurse, and have not had a reply from a single candidate fulfilling the requirements of the Local Government Board. The Guardians have, therefore, asked that a well recommended woman, but not technically qualified, might be appointed. Very much might be done to ease the present tension by co-operation, or by a system of combined certificates. There are many institutions of a more or less special character that give certificates for a longer or shorter period, but under the stipulated three years. It would be of little use to give three full years' training in any institution that was not a general hospital, but certificates showing a training of one or more years' at a general hospital, one or more years' at a special hospital—that is, at a fever, eye, ear, or gynæcological hospital—and one or more years' experience in a large Poor-law infirmary, might be welded into one three-year combined certificate qualifying for the superintendent's post and accepted by the Local Government Board. This would not lower the standard at present required, but it would technically qualify many nurses who have excellent credentials, both of good training and of having filled important posts as charge nurses, or sisters in efficient training schools, but whose certificate from any one hospital is for a shorter period than the three years.

### "EASTWARD HO!"

THIS is the title of a bright little monthly journal that is published by the Poplar and Stepney Sick Asylum, Bow. This is noteworthy on the score that it is the first periodical published by a poor-law institution. This asylum is one of the largest in London, and contains 770 beds. It is not so very long since it became an established training school for nurses, but its graduates are already taking a very good place in the nursing world. The founding of the little paper is due to the energy of some members of the nursing staff, who are endeavouring by this means to keep alive the spirit of good fellowship engendered by studying and working together. The aim is excellent, and the manner of carrying it out attractive, and we trust "The Hobby-horse of the P. and S.S.A." (the sub-title) will attain popularity, and remain a bond of union between those who have worked and are working in the asylum.

### AN ANECDOTE.

THE following little story of the indiscriminate zeal of the amateur nurses in their attention to the wounded in the Zulu war has recently appeared in *Madame*. It is meant to be very funny, but is possibly not without a grain of truth. Amateur Nurse: "What can I do for you, my poor fellow?" Wounded Man: "Nothing, thank you, miss." A. N. (persuasively): "Not *anything*." W. M.: "I don't think so." A. N. (mildly, but firmly): "I can wash your face." The ablutions performed, she exclaims with satisfaction: "There, now you feel nice and clean!" W. M. (with a weary smile): "I ought to, miss! You're the ninth lady who has washed my face this morning!" A most patient patient, truly! The majority of sick men would have said some short, naughty words at washing number two.

### GIFTS TO THE BRADFORD NURSES' HOME.

VARIOUS handsome gifts have been made to the new Nurses' Home at Bradford. The chairman has given an old grandfather's clock in a carved oak case, which has been placed in the entrance-hall. Mr. Frederick Illingworth has presented complete sets of the works of Dickens, Scott, and George Eliot, which have been put in the nurses' study. Other ladies and gentlemen have given pictures and a barometer.

### SHORT ITEMS.

THE sum of £63 resulted from the recent charity ball held at Stockton. One-third of this amount goes to the Nursing Association.—What might have been a fatal accident happened at Blackpool. Nurse Todd and her patient were walking on the pier, when the latter lost her balance and fell into the water. The nurse immediately jumped in after her, and both ladies were fortunately rescued by two fishermen. Nurse Todd showed great courage and promptitude.—The Countess of Aberdeen has consented to become president of the Woman's Industrial Council. The following ladies have promised to lecture on behalf of the funds during February and March: Miss Maud Phillips, Mrs. Bosanquet, Mrs. Hogg, and Miss Honnor Morten.

### Hints on the Home Nursing of Sick Children.

By J. D. E. MORTIMER, M.B., F.R.C.S., formerly Surgical Registrar, &c., at the Hospital for Sick Children, Great Ormond Street.

(*Continued from page 191.*)

### MASSAGE—ADMINISTRATION OF MEDICINES—ENEMATA.

### MASSAGE

may be required for its local effects, as in chronic constipation or infantile paralysis, or for its general effects, as in convalesence after acute illness, nocturnal restlessness, and certain stages of chorea. The subject cannot be dealt with adequately in this limited space, but I would say that with children it should at first be only done for a few (say, five) minutes, and without approach to roughness. If the nurse does not leave off *before* the child is tired, or if she causes pain, there will be difficulty in beginning next time. It is a good plan to engage the attention by telling a story or singing whilst at work. These remarks also apply to treatment by electricity. The child, if nervous, should be induced to regard the battery as a plaything, and the electrodes should be at first applied without turning on any current at all.

### ADMINISTRATION OF MEDICINES.

We must not forget that giving medicine is only one part (and sometimes not a very important part) of the treatment (or rather, the management) of a case of illness, and that if there is strong repugnance on the part of the child, or a fight over every dose, it may be wiser to discontinue, lest we do more harm than good. Before the medicine is offered this question should have been decided, so that if objection is made the nurse may act as may be best under the circumstances. If the child is only distrustful a little coaxing may be all that is needed, or the nurse, having tasted the medicine, may be able to give an assurance that it is not nasty; but if this is not the case the child should be frankly prepared for an unpleasant taste and encouraged to be brave. With small children it need not be undignified for the nurse to put a favourite doll under the same course of treatment! Untruthfulness, bribery, or threats should never be resorted to. If, however, the refusal is simply due to obstinacy the child may be good-humouredly told that he is a silly boy or that it is not worth while to make him take it if he prefers to be ill, and the medicine may be put away for awhile, but if the doctor considers it essential that it should be given, and if the parents do not object to the employment of force, little time should be wasted in argument or entreaties. One or two assistants should be called. The nurse should have the child lying across her lap, whilst one assistant (on her left) holds the head firmly by putting the palms of her hands flat on each temple, and the second assistant (on the nurse's right) holds the child's hands down on his thighs, putting the legs between her knees, with the edge of her chair against them to prevent kicking. The nurse then pinches the nose, and with the little finger of the same hand in the corner of the mouth hooks out the cheek, so that from a feeding cup held in her other hand she can pour the medicine in between the cheek and the teeth, or inject it from a syringe fitted with a piece of tubing. The fluid sinks down to the back of the mouth, and the child after a moment or so is obliged to swallow it, as he cannot breathe through his nose. If there is only one assistant the child must be laid across the bed with the legs hanging over the side. The assistant takes the arms and legs as before, whilst the nurse kneels on the bed and holds the child's head between her knees. In such cases firmness is the truest kindness. A prolonged fuss does much harm all round. The lesson is also likely to have a good effect on the child's morals, although I do not think illness is the time when efforts should be made to atone for a previous want of proper discipline, especially in such cases as uncomplicated pneumonia when medicine is least necessary and generally most disliked.

Most drugs can now be ordered in a pleasant form. Some may be given tastelessly in milk or broth. Powders may be given to infants by taking them up on the finger or feeding teat moistened with glycerine and letting the baby suck them off. Older children will take them in a spoonful of bread and milk or a bread and butter sandwich. If the medicine is bitter, sugar or a peppermint lozenge may be given beforehand. Other tastes are lessened by holding the nose when the dose is taken. If the mouth is dry and hot it should be rinsed with cold water beforehand.

It is often advised to shake cod-liver oil or castor oil with warm milk flavoured with cinnamon. This may, however, cause a dislike to milk itself. These oils may be taken in capsules by older children or may be made into an emulsion (Mellin's is stated to contain 50 per cent. or half cod-liver oil). Cod-liver oil may also be given with sardines, and there are numerous combinations with malt extract, which, however, are not very satisfactory either as regards palatability or the amount of oil contained in them.

Leeches should, if possible, not be seen by the child. They may be taken up in cotton-wool, or the box turned over on the skin.

### ENEMATA.

For relief of constipation a glycerine suppository or "enule," or injection of a teaspoonful of glycerine will probably be sufficient. If a syringe with a shoulder* cannot be procured, the finger must be kept on the nozzle half an inch from the end, so that it cannot do much harm if the child suddenly moves. When a child strains and cries much without result, the interior of the bowel should be examined with the little finger, and if hard lumps are felt some warm oil should be injected. It may be even necessary for the nurse to remove an accumulation. This should be done with the finger only—the responsibility of using any kind of instrument should be left to the doctor. Large enemata may be needed to wash out the bowel for removal of worms or in certain diseases, and should be given very slowly with a Higginson's syringe, or preferably with tube and funnel (not raised more than two feet above the bed). It is better to desist if there is much straining and struggling. Small enemata of thin gruel (an ounce or two) are useful when the lower bowel is in an irritable state.

I may mention that a tingling scarlet rash (like that of scarlet fever, but unaccompanied by sore throat, &c.) may follow the administration of an enema or aperient, especially if there has been much constipation previously. Nutrient enemata have already been mentioned.

* Rogers, 327, Oxford Steeet, W.

(*To be continued.*)

### Sutton Cottage Hospital.

SUTTON has just come possessed of a cottage hospital for six beds for the large rental of one shilling per annum. The building was formerly known as the Sutton Infectious Diseases Hospital, but the District Council no longer utilising it as such, they handed it over to the people of Sutton to be used for cases of general illness and accidents. Mrs. Bucknill opened the hospital; and, at the same time, substantial donations and subscriptions were announced.

### Grand Canary as a "Winter Health Resort."

Having lived and worked in Grand Canary for the greater part of the last three years in the double capacity of patient and nurse, and having benefited thereby greatly from the health point of view, it seems to me well worth while to publish some of the practical results of my experience in the hope that other invalids may be induced to follow my example, and take flight southward when they find the cold, damp English winter too trying for their delicate lungs or aching limbs.

Grand Canary is the largest and most important commercially of the group of Canary Islands lying off the West Coast of Africa, about a day's sail south of Madeira, and its capital is Las Palmas, in the English quarter of which city I lived for the greater part of my time abroad.

The drawbacks to a sojourn in this sunny climate are so few in proportion to the advantages that we will consider them first. First, it is too far away from England for very ill English people to go to unless they can arrange to take friends and nurse with them. I have seen sad cases of invalids arriving in a rapidly advancing phthisical condition, with scarcely strength to rally from the fatigue of the voyage, needing day and night nursing, and missing the kindly attention of home relatives and friends. Second, the semi-tropical climate naturally causes some discomfort to English visitors from the increased small animal life which it always encourages. Mosquitoes, flies, &c., are somewhat troublesome in the summer, and in the Spanish Fondas and dwelling-houses, some of which latter are rented by English residents; but in the winter season and in the good hotels these small pests are rarely met with. Third, the journeys to and from Las Palmas and the living when there are all expensive. The island does not produce much in the way of fish, flesh, fowl, and other delicacies easily obtainable in England; these are imported, and therefore dear and scarce, except at the good hotels. Fourth, occasionally in very dry weather the fine sand is driven by the wind and traffic along the main roads in such a way that those with delicate breathing apparatus are advised for the time being to remain either within the hotel grounds or on the low-lying hills immediately behind them.

And now let us consider some of the advantages which according to my way of thinking so far outweigh the aforesaid drawbacks: First, to counterbalance the poor quality of butchers' meat there is an abundant supply of delicious fresh fruit, oranges, bananas, apricots, peaches, cherries, grapes, figs, &c., all good and cheap, and always one or the other in season. Second, the climate all the year round is warm, dry, and equable; even at night the temperature seldom falls below 60 deg. throughout the winter, and then there is so little damp in the air that it is usually quite safe to sleep with open windows. The rainfall is very small, and occurs almost entirely between October and February; occasional heavy showers (generally in the night) are quickly followed by bright sunshine, blue skies, and the prevailing north-east wind, which is decidedly exhilarating; every one grumbles when on rare occasions the breezes come from the south; then only the English folk feel somewhat depressed, and the Spaniards go out muffled up in great coats and scarves, but this does not often happen. The lightest summer (English) clothing is habitually worn in Las Palmas by our fellow country men and women all through the winter, a warm wrap only being needed for the return drive from a long day's excursion up amongst the mountains. Third, amusements abound for patients' friends and for those patients who are well enough to take part in them; golf, cricket, tennis, and croquet. The hotels give numerous dances during the season, and are quite gay when English men-of-war are in the harbour. Fourth, there are two first-class hotels under English management, which form, as it were, a nucleus round which is chiefly gathered the English part of the population of Las Palmas. The Hotel Metropole, originally a large dwelling house built after the Spanish fashion round a patio, has been considerably added to, and is liked by many people because of its situation close to the sea, but its other side is on the main road, which is at times both dirty and noisy. The Hotel Santa Catalina is a picturesque building, with many verandahs and flat roofs, standing within extensive well laid out grounds, within easy reach of the beach, but just out of the way of the noise and dust of the road. This hotel was expressly built for the accommodation of English visitors about eight years ago and is admirably adapted for the purpose, one of its chief recommendations being its perfect sanitary arrangements. This has been my Canary head-quarters where I have always stayed between my cases, and where I have always been very happy and comfortable, and where I have several times been most kindly cared for when I was over-tired or ill. The manager's wife is herself a trained nurse, and is of a most thoughtful, tactful disposition, which contributes largely to the comfort and well-being of guests staying in the hotel. The garden is delightful—roses, honeysuckle, and many other sweet flowers which we are accustomed to at home, together with many semi-tropical plants, blossom luxuriantly during the winter and spring months; there are tennis and croquet grounds, and a golf links; sheltered walks and cosy corners for the delicate ones, and many other seats and promenades in the grounds for the more robust to sit or take exercise in. There are bedrooms of different sizes and with different aspects to suit the various purses and constitutions; all are exquisitely clean and sufficiently well furnished. The public rooms are large and comfortable, or private sitting-rooms can be had if necessary. The charges in both hotels are about the same, from 10s. 6d. a day, rising according to the accommodation provided. Fifthly, each hotel has a resident trained nurse, who is charged for according to the service required of her. Sixth, an English physician has a house close by, and spends a great part of each day in the hotels. He has lived in Las Palmas for about eight years, and so has had a long experience of the climate and amongst those invalids who generally go there. Patients who immediately after arrival put themselves in his hands and adhere strictly to his instructions invariably make better progress than either those who lay down their own rules or those who try to regulate their proceedings according to the instructions of their home medical adviser. Seventh, near to the hotels is an English church, to which a chaplain is appointed each winter for the season. Eighth, some phthisical patients benefit greatly by staying two winters and the intervening summer in Grand Canary. For their accommodation in the summer, when Las Palmas is apt at times to feel somewhat airless, there are two English hotels in the mountains, about an hour's drive from the city, in the midst of the vineyards (where delicious grapes may be purchased at the rate of one penny a pound), and surrounded by beautiful scenery.

There are shops in Las Palmas, but they are practically useless for the English visitors; it is wiser to take out everything—clothes, books, needlework, drugs, &c.—which is likely to be needed.

The Canary Islands belong to Spain. It is a source of interest and amusement to watch the habits and customs and to learn the language of another nation so long as your bodily necessities and comforts are all provided for in your hotel; but if you determine to set up housekeeping on your own account in Las Palmas you will soon decide that you never want to have anything more to do with a Spanish servant.

The tariff of charges, &c., of the Santa Catalina Hotel may be obtained from the Canary Islands Company, 1, Laurence Pountney Hill, E.C.; that of the Hotel Metropole from Messrs. Elder, Dempster, and Co., Liverpool.

How to Reach Las Palmas.

From the North of England it is easier and considerably cheaper to go from Liverpool by one of Elder, Dempster's boats, which sail every Saturday, and take a week for the voyage; £15 return first class. But much more comfortable and also more expensive are the Castle boats, leaving Southampton once a fortnight, and reaching Canary in five days. For good sailors the Forwood line of boats from London are satisfactory, for they go by the Morocco coast and Madeira and so make an interesting journey, but they carry no doctor, and are therefore unsuitable for invalids. A useful handbook is the one published by Samler Brown under the name of "Madeira and the Canary Islands." The patients that I have seen do best in Las Palmas are: (1) Cases of phthisis in its early stages; (2) cases of convalescence from severe pneumonia, pleurisy, and other acute lung affections; (3) cases of rheumatism; (4) in cases of far advanced phthisis the disease is sometimes arrested in what appears to be a marvellous manner, and patients are enabled to live there a fairly active outdoor life who would be wretched bed-room invalids nearly all the year round at home. I believe that the climate also acts beneficially in many other ailments.

### Training in the Provinces.

(*Continued from page* 192.)

### THE COUNTY HOSPITAL, YORK.

Terms of Training.

Candidates for training at the York County Hospital should be between 20 and 30 years of age, and well-educated women. The period of training is for three years, candidates entering in the first instance upon a month's trial. If accepted at its expiration they pay a fee of £20, and are supplied by the hospital with full indoor and outdoor uniform. Laundry is also provided. No salary is paid to probationers during their first year. For the second year they receive £12, and for the third £16. Sisters' salaries range from £20 to £30 per annum.

Lectures are given to the nurses by members) of the honorary and resident staffs, and instruction in sick-room cookery is also provided. Certificates of training are only given on completion of the three years' examinations, for which prizes are offered, being held during the course. All nurses are alike eligible for promotion to the post of ward sister on completion of their training if they have passed their examinations with credit; but there are not often vacancies, and no provision is made for nurses remaining in the hospital beyond the three years unless they are so promoted. Night duty is taken for three months in each year by the nurses, working under a permanent night sister.

Hours On and Off Duty.

Probationers are on duty in the wards between 7.20 a.m. and 8.30 p.m., with time for meals and an hour and a quarter daily for recreation. They have a half-day once a week, from 2 to 8.30 p.m., and from three to five hours on Sundays. Sisters are on duty from 8 a.m. to 8.30 p.m., with time for meals, and their time for recreation is the same as the nurses. All the nursing staff have a calendar month's holiday during the year, often extended to five weeks, and they are allowed to divide the time and take a fortnight at the end of the first six months if desired. The night sister gets five weeks in the year, with a night off each month and some day duty if desired.

Meals.

Sisters and nurses use the same dining-room for all meals except lunch, which they have in the day-rooms off the wards. All food is provided in the dining-room for each meal, except for the early lunch, for which stores, consisting of tea or cocoa, jam, biscuits, or cheese, are given out weekly to each ward day-room. The night nurses have a meat meal provided in a ward day-room, as the dining-room is too far from the wards to be used at night. A special cupboard is provided for keeping these stores. The hours of meals are as follows: Nurses' breakfast is at 7 a.m.; lunch, 9.30; dinner, 1 p.m.; tea, 4.20; supper, 8.30 p.m. Sisters' breakfast at 7.45; their lunch is at 12.30; tea, 5.30; and supper at 8.30 p.m. Night nurses have tea when they are called at 7 p.m., their breakfast is at 8 p.m., and dinner at 8.30 a.m., while they have two meals during the night, one at 12 and another at 4.30 a.m.

### Appointments.

The Royal Hospital for Children and Women, Waterloo Bridge Road, S.E.—Miss Katherine Maud Moore was appointed Matron of this hospital on February 7th. She was trained at St. Bartholomew's Hospital, and has been staff nurse and ward sister at the Hospital for Sick Children, Great Ormond Street, and afterwards superintendent of the convalescent branch of that hospital. She has also been superintendent of the infirmary of the Foundling Hospital.

Barnsley Workhouse Infirmary.—Miss A. Florence Shedden, who was trained for sixteen months at the Guest Hospital, Dudley, and for three years at the Workhouse Infirmary, Birmingham, was appointed Lady Superintendent of the above hospital on January 26th. Miss Shedden was for eighteen months engaged in private work; for twelve months she was charge nurse at South Fulham Fever Hospital; and afterwards charge nurse at Paisley Abbey Hospital, and at Craigaith Hospital, Edinburgh.

North Staffordshire Infirmary and Eye Hospital, Stoke-upon-Trent.—On January 12th Miss E. J. Jessop, who was trained at the General Infirmary, Leeds, was appointed Superintendent of Nurses here. For the last eight years Miss Jessop has been matron of the Grimsby and District Hospital.

The District Hospital, Newbury, Berks.—On January 27th, 1899, Miss A. M. C. Bewkitt was appointed Matron of the above. She was trained at the Royal Infirmary, Edinburgh, where she afterwards became nurse.

### Royal British Nurses' Association.

The eleventh annual conversazione of the Association will be held on Monday next, February 13th, at half-past eight p.m., in the rooms of the Royal Institute of Painters in Water Colours, Piccadilly. A most attractive programme has been arranged, and the railway companies are offering, as before, facilities to country members by the issue of return tickets at single fares on production of the card of invitation at the ticket office. Applications for tickets for the conversazione should be made to the Secretary, 17, New Cavendish Street.

Lectures.

The following lectures will be given at the Rooms of the Medical Society of London, 11, Chandos Street, W., at six p.m.: Friday, February 10th, "The Nurse's Part in the Prevention of Tuberculosis," by Dr. Bezly Thorne. Friday, February 25th, "Our Vaccination; the objections made to it, the causes of failure, its history and essential nature" (illustrated diagram); by Mrs. Garrett Anderson, M.D. Friday, March 24th, "Sanitary Science for the Nursing Profession," by Mrs. Clare Goslett. Members are invited from five to half-past seven, and may bring a friend, without charge for admission. Tea and coffee will be served in the committee room between five and six o'clock.

### In an Indian Hospital.

(BY A CORRESPONDENT.)

### THE CONTAGIOUS WARDS.

OUR contagious wards are called into requisition to hold cases of measles, small-pox, and cholera in turns, and, horrifying as this must sound to English ears, no harm has arisen from this practice during the seven years in which I have nursed in them. Of course, one could wish for some better arrangement, but as it is apparently impossible to alter this state of affairs at present, we do all we can, and leave the rest. These wards are in one block, built of stone; they consist of four large rooms, all opening on to a verandah, which runs the length of the building, each supplied with a good-sized bath-room. We have also two large tents for these cases, as we are very pushed for room sometimes.

Infectious diseases seem, at least in this part of the world, to have their special seasons: in the spring measles is prevalent, in the hot weather small-pox, and during the rains cholera. These widely differing diseases are at present of necessity nursed in the same set of wards and tents. When one of these is empty it is disinfected in the following manner: The walls and ceiling are well washed with strong hyd. perchl. lotion, and the stone-flagged floor wiped with strong (usually the crude) carbolic. I have lately been presented with a pump, which will simplify matters a great deal. While the room is still dripping wet the doors, windows, and ventilators are closed, and lump sulphur burnt for twenty-four hours, after which it is then opened up for use.

Of furniture there is practically none, for the natives use webbed cots, on which I have often slept myself, and for the comfort of which I can answer. We have also two spring beds for European cases, and by the side of each a rug is spread. There are also two small tables and three chairs, which have to do duty for all the European cases. Fortunately there are seldom more than two of them at a time. Crockery also is limited, and varied in style.

Bed-linen and clothing are still a source of constant anxiety as regards the native patients, especially during the cholera season, and, of course, many things have to be burnt. Until two years ago, we were almost as badly off for clothing, &c., for Europeans. The pillows and mattresses (the latter are very seldom used, the patients preferring the plain webbed cots) for the native patients are made of coarse drill, filled with straw, and as each patient goes out of hospital or dies, these drill cases are opened, the straw burnt, and the covers, after having been soaked in lotion and exposed to heat, are sent to the wash, to be refilled when they are returned. The pillows for Europeans are filled with a kind of horse-hair, which, after use, is soaked in lotion, and exposed to great heat; it is then ready for the next patient. One must see personally to the disinfecting of all linen, clothes, &c. Until this year, my medicine was dispensed mostly in one-pint beer bottles, my ointment in Liebig pots, and the general mixtures in brandy bottles. However, it answered all purposes, and that was the chief thing. I am the happy possessor of one spatula, which performs several duties. The glass windows and ventilators, which are numerous, are all painted green. We have a light wooden stretcher, kept always in the verandah of this ward, which is frequently scrubbed down with lotion and hot water. In the hall of the admission department of the hospital there is a webbed one, covered with a macintosh. When the servants on duty have to bring a case from the hall to the infectious ward, they will very often use the webbed stretcher rather than come and take the proper one in spite of the trouble this entails of taking off the webbing and re-taping it again.

During the last cholera epidemic, which was unusually severe, a fresh student was appointed every week to help me with the dry-cupping and hypodermic injections, and to be shown the general work of the ward. After the students have passed their third year examination, they are often sent to cholera camps. Most of them were very willing to learn all they could, working admirably, asking to be allowed to help to make the mustard plasters, apply friction, and hot fomentations, and inquiring the why and wherefore of everything. They were all much interested in the cooking of Benger's Food, and surprised at the care necessary to make it of any use. But I had to ask for two of them to be removed, as they were in a state of abject terror. During a bad cholera season this ward is far from quiet, what with delirious patients, the incessant vomiting and hiccuping, the smash of hot water bottles (I have only ordinary glass ones), and the violent grief of the relations when anyone dies.

Then there comes the bright side. Gradually some of the patients safely turn the corner to convalescence, and one morning their bed linen is taken out and spread on the verandah, and they are brought out for an airing, after which they are very unwilling to be taken back to the wards. It is usually during these morning airings that the different members of the same family meet—mother and sons, &c.—for we frequently have whole families brought in together. At length the day comes when the patient is declared fit for discharge, and, after the last disinfectant bath, gets into his clean clothes and goes to his home, well pleased with the whole world. An ungrateful patient is a very rare thing, at least in my experience. Occasionally there is great difficulty in injecting and cupping a new patient. The great thing (if the new-comer can be sufficiently calmed to understand) is to get a convalescent to show him the marks of the injections on his own body. This they are always more than willing to do. It is characteristic of cholera that in the early stages of convalescence the feeling uppermost in the mind of every adult is that it would have been so much nicer to have been allowed to die—indigestion, and a tendency to hysteria, very often follow an attack.

During a small-pox epidemic many of the patients are able to get about to a certain extent for a great part of their long convalescence. In the evening the surrounding compound then presents a strange sight, when the sun has gone down, of people walking about in very grotesque costumes, blankets folded round them or shawls. The little ones whose feet are too sore to allow of their running about are carried by turns. It is then one of the merriest wards of the whole hospital. Both native and European patients, of course, look very quaint, with their faces all light pink paint, and just their eyes peeping out. Once I had just painted and oiled a tiny native baby about three years old, and had set him on the floor to dry, with a red fez cap on his head by way of clothing, when a dog passed by, and at once stopped and began barking furiously. Baby, highly delighted, tried to get out to the dog, which had at last to be forcibly removed, for we were afraid he would fly at the child. The morning visit of the doctors was just going on, and the occurrence excited great amusement.

I have known European patients start back on first seeing one another, each unable to believe that he himself presented quite as odd an appearance as his companion. I found one once with the largest brown teapot the ward boasted of trying to find out what his face looked like. The men are always much pleased when their lips allow them to whistle once more. It is a most rare thing to have a death from small-pox, or to have the patients pitted if they come in fairly early. The natives bring patients willingly of their own accord, though during the plague time they hid them from fear of their being sent to the plague hospital whilst the premonitory fever lasted. This year not a single cholera case has come in; such a thing has not been known for the last eight years. I can only put it down to the thorough cleansing the city underwent during the plague epidemic.

### Novelties for Nurses.

### THE "HAPPY THOUGHT" COT PROTECTOR (PATENTED).

To prevent falls from the cot, which are a frequent cause of injury to children just beginning to walk and climb about, is the object of an invention by Mrs. Murphy. It consists of a light frame of bamboo and netting, which can be easily fixed to the cot and removed at pleasure. The child is thus prevented from falling out, while it can play about freely on the bed if awake and restless when its mother or nurse is still asleep. The frame is inexpensive, and can be folded up in a small space when not required, and is to be had of the chief furniture makers.

### WARM SLEEVELETTES.

The Knitted Corset and Clothing Company, of 118, Mansfield Road, Nottingham, are supplying a most welcome novelty in the form of a warm knitted sleeve which they call the "Cosy Armlet." Such an article has really been needed for some time past. To supply the deficiency we know it is a common practice with many people to utilise old stockings for the purpose of an extra covering for evening wear under cloak or cape, or when the weather is very cold under the sleeve of the dress. But such a contrivance is only a makeshift and has the attendant inconvenience. The stockings are not made with fitting terminations at the top and at the wrist, as in the case of the armlet before us, and so they slip down, become uncomfortable, and do not serve the purpose intended. The Nottingham company have realised what was needed and have prepared an article of clothing which no woman should be without. Many chills and rheumatic pains will be avoided by the use of these simple and convenient armlets. They slip on and off in a moment and are so soft and elastic as to cause no inconvenience to the wearer. The Knitted Corset and Clothing Company will send a list of all their useful garments free on application.

### A NICE JELLY.

(Sutcliffe and Bingham, Ltd., Khovah Works, Manchester.)

Jelly is a very useful addition to the diet of a sick person, but at the same time nurses often refrain from suggesting it for their patients owing to the fact that few cooks understand the art of jelly making. In their Khovah Jelly, sold in large tablets, Messrs. Sutcliffe and Bingham have removed all difficulty. The jelly is guaranteed to be made of pure gelatine and fruit juices, and is not only wholesome, but really delicious in substance and flavour. The making process is solely one of dissolving in hot water, so that a nurse may prepare the jelly herself in a few minutes if she will. For the private nurse, the district nurse, and as an extra in the ward kitchen, it will be found equally acceptable. Matrons also will find it serve as an economical variety for the table of the medical or nursing staff.

### Minor Appointments.

St. John's Workhouse, Hampstead.—On January 26th Miss Margaret Beauchamp Nuttall was appointed Night Sister of the infirmary wards. She was trained at Addenbrooke's Hospital, Cambridge (three months), at the Royal Infirmary, Manchester (two months), and at the Ancoats Hospital, Manchester (two years and seven months). She has been for the last fifteen months charge nurse at the Hampstead Infirmary.

South-Eastern Fever Hospital.—Miss Annie McGowan was appointed Charge Nurse at the above hospital on January 11th. She was trained at, and afterwards became staff nurse at the Poplar and Stepney Sick Asylum, and since then she has for two years and nine months been at the National Hospital for Consumption, Ventnor.

Swansea Hospital.—Miss Helena J. Mooney was appointed Sister of the Pellergan ward, operating theatre, and out-patient department here on January 1st. She has had 18 months' training at the Salford Fever Hospital, and 15 months' at the Ships, Dartford, and three years' general training in this hospital as probationer and staff nurse.

St. Saviour's Infirmary, East Dulwich Grove.—Miss Frances Bruce Boutchart, sister at the above infirmary, has been appointed Night Superintendent. She was trained at the Royal Infirmary, Dundee, and was subsequently sister at tho Royal Infirmary, Wigan.

Shields Union Infirmary.—Miss E. Clements informs us that she was Sister of the Male Surgical Pavilions at Poplar and Stepney Sick Asylum, and not charge nurse as stated in the notice of her appointment to the above infirmary in our last issue.

St. John's Hospital for Skin Diseases, Uxbridge Road, W.—Miss Annie Ellis was appointed Sister of the female wards here on January 31st. She was trained at the Warrington Infirmary, where she afterwards had charge of the medical and surgical wards for two years.

Oldham Union Workhouse.—Miss Letitia Middleton was appointed Charge Nurse here on February 1st. She was trained at the Hope Hospital, Salford, and was afterwards charge nurse at Haslingden Workhouse Hospital.

Royal Portsmouth Hospital.—Miss E. Brown, who was trained and afterwards sister at the Warneford Hospital, Leamington, has been appointed Sister of the above hospital.

### Pension Fund Nurses.

### MISS BURNS' WEDDING GIFT.

We have to acknowledge receipt of two more contributions, from Nurses Quiggin and Daniel.

### A Book and its Story.

### THE LAUREL WALK.

In the depiction of quiet home life Mrs. Molesworth has few rivals, and a new book* by this popular authoress is a welcome change from the highly-coloured fiction so common now-a-days. However slight the plot may be, her characters have always a keen interest for her readers, for they are life-like sketches of the people we meet every day. The plot of "The Laurel Walk" is a simple one. It is the story of a younger branch of a family living in straitened circumstances under the shadow of the "big house" to which they believe they are entitled under the terms of a missing will. The will is found eventually under circumstances which give the title to the story, and then, according to the usual custom of novel writers, everything should end happily, but Mrs. Molesworth's dénouement has other surprises, which we will leave our readers to find out for themselves.

The story opens with a description of the Morion family. Mr. Morion, a querulous, conceited, self-centred, and selfish hypochondriac and a nuisance to his family, who he bullies. Lady Emma, his wife, a somewhat weak character, crushed out of all entity by her husband. "She was a good woman, and meant to be, and believed herself to be, an excellent mother, but under no circumstances in life could she have fulfilled more than one rôle, and the rôle which she had adopted since early womanhood had been that of wife." The type is a common one. She "was not a woman of much intellect or, what matters more in a mother, of any width of sympathy." She consequently does not see how hardly the lack of all brightness falls on her daughters, and makes no attempt to bring any interest into their lives. Her three daughters consequently live dull lives, their acquaintances are few, and they see nothing of the society with which their birth entitles them to associate.

Frances, the eldest girl, the mainstay of the family, who is depicted as a fine character, is the real mother of the two younger girls, and her attitude towards them is well indicated in the opening chapter. Betty, the second daughter, is complaining of the emptiness of their lives. "Perhaps so," Frances agreed, "but it isn't only circumstances that make lives. There are people far poorer than we, I know, whose lives are ever so much fuller and wider. It is that," she went on speaking with unusual energy, "it is that that troubles me about you two! I want to see my way to helping you to make the best you can—in the widest sense of the words—of your lives," and her sweet eyes rested with almost maternal anxiety, pathetic to see in one so young, on her two sisters. "And you, you poor old darling," said Eira; "what about your own life?" "Oh!" said Frances; "I don't feel as if I had any separate from yours. All my day dreams and castles in the air and aspirations are for you." Betty, the impulsive second sister, is a delightfully natural character, and Eira, the youngest sister and the beauty, who "thinks life would be quite a different thing, twice or three times a interesting" if she only had "heaps and heaps of beautiful clothes," is also a charming study.

What adds to the grievance of the Morions is that their cousin, the legal proprietor of Craig Morion, never occupies it, and it has remained empty for years. With the opening of the story, however, new tenants come to the 'big house' in the shape of Horace Littlewood (the brother-in-law of Ryder Morion, the proprietor), and his mother and sister. Horace is an officer, on sick leave from India, from the effects of a fall at polo, and it is round him that the love interest of the story is woven. The two younger girls at once imagine a match between him and Frances, the elder sister, and Frances, ignorant of their wishes, falls more or less in love with him. His affections, however, are fixed upon Betty, who, in spite of her plans for her elder sister, falls in love with him herself, and the way in which she conceals her feelings is cleverly and touchingly worked out. The course of their love does not run very smoothly, for Horace is dependent on his mother, and she wishes him to make a wealthy marriage, and will not hear of the match. Mr. Morion, also when he finds that Mrs. Littlewood will not finance the young couple, withdraws his consent, and matters have apparently come to a deadlock when Mr. Ryder Morion comes to the rescue like a good fairy, and everything ends happily. Frances' not very deeply engaged feelings find a much more suitable object than Horace Littlewood, and the book closes with her as mistress of Craig Morion.

All the characters in the story are very skilfully drawn, perhaps the cleverest being Mr. Morion. Soured by want of means, peevish, fretful, and petty-minded, he is for ever standing on his dignity, and on the look-out for slights, and still more for people attempting to patronise him. Altogether, he is one of the most irritating characters we have come across in fiction for some while. His whole character is well shown in one of the scenes. Horace Littlewood has asked the girls if Lady Emma will be at home, as he hopes to call on her."

"He asked me if he might call to say good-bye to you, mamma, to-morrow afternoon." The last words, unfortunately as it turned out, were overheard by Mr. Morion as he entered the room. His wife, taught by long experience, made no reply. So the message remained uncommented upon, unless a doubtful grunt from the depths of the arm-chair, where the master of the house had settled himself, could have been taken as referring to it. Mr. Morion's 'den,' as in jocund moments he condescended to call it, opened, unfortunately, on to the hall, almost opposite the drawing-room. In some moods he had a curious and inconvenient habit of sitting with the door open, and though he sometimes complained of advancing years bringing loss of hearing, there were times at which his ears seemed really preternaturally acute; and this afternoon, thanks to this peculiarity, aided possibly by some occult intuition of anticipation in the air, he was somewhat on the *qui vive* for—he knew not what. Suffice to say, he was in a rare state of nervous irritability, ready to quarrel with his own shadow could that meek and trodden down phantom have responded to his need. As Frances was handing him his cup of tea the front door bell rang. A thrill of expectancy passed through Betty and Eira. 'Who can that be?' said their father, in a tone of annoyance. 'It is probably Mr. Littlewood,' said Lady Emma quietly, 'calling to say good-bye. I was expecting him.' 'Very strange, then, that you did not mention it to me,' replied her husband acridly. 'Am I in a fit state of health to be troubled with visitors to-day? Not that it signifies; he need not be admitted.' 'Papa,' said Frances, in a tone of remonstrance, 'it will seem very rude—he asked if he might call—we met him yesterday, and . . . .' Mr. Morion rose from his seat, and opening the door, gave his orders in a decided voice. 'Parker,' he said, 'if that is a visitor, say at once that her ladyship is not at home, and that I am not at home either.'"

* "The Laurel Walk." Mrs. Molesworth. (London: Isbister and Co. 1898.)

### Everybody's Opinion.

[Correspondence on all subjects is invited, but we cannot in any way be responsible for the opinions expressed by our correspondents. No communication can be entertained if the name and address of the correspondent is not given, as a guarantee of good faith but not necessarily for publication, or unless one side of the paper only is written on.]

### NO LONGER ABLE TO WORK.

"E. T." writes: May I offer a few suggestions to middle-aged nurses, so many of whom are no longer able to find employment? Firstly, I strongly recommend the Pension Fund. Nurses can, and ought, to save during their best years. It can be done, I know ("Where there is a will there is a way"). Secondly, I for one should appreciate a nurses' settlement or almshouses. I feel sure each nurse would prefer a bed-sitting room in a permanent home; but for members who are very poor perhaps cubicles would be found more economical. Then there are many very excellent homes of rest and convalescent homes, which, besides being moderate in their charges, offer reduced railway tickets. Such places are all in our favour. I am only advising the thrifty nurses; the others are hopeless. I would also like to caution nurses against the Superannuation Act. Of what use will that be to them if they try to change situations after 45 years of age? It is hardly possible at that age for them to get another post in the service, so that what they pay will often be lost to them. It should be made a safe and certain pension for all who join it if they keep up their payments, though, no doubt, there will be more for those at the top under the present conditions. Poor nurses should not rely on that at all, but endeavour to help themselves in some safer way.

"Sympathetic" writes: Maternity nurse wishes to suggest to "Miserère" and others who find themselves displaced by younger nurses that it might be worth their while to take up nursery work. The demand for trustworthy women to take charge of the young far exceeds the supply. Maternity nurses and those who have trained in children's hospitals would be specially suited to it. The work, though responsible, is usually pleasant and cheerful, and not heavy. Also to take a baby from the month is work more suitable for a woman of experience than for a young girl, who is all right for under-nurse when the little folks increase in numbers. The nurseries are generally pleasant rooms, and the nurses get open-air exercise daily, weather permitting. The pay is anything from £20 upwards. Surely that is better than weary waiting for work which does not come, and spending the savings which will be so sorely needed by and bye. The care of the rising generation is of such high importance that no womanly woman need feel it beneath her. To the younger nurses I would also say a word. In a few years their turn will come to be ousted by "younger nurses," therefore let them take warning and make provision for the future now they are able, by paying in early and regularly to the Royal National Pension Fund, which was not started when we were young; and then they can see themselves passed by with more equanimity than can the older nurses now.

### A BOLD COURSE OF ACTION.

"A Correspondent" writes, in reference to the paragraph which appeared in our last issue with the above heading, as follows: In defence of the manner in which the nursing staff of the Altrincham Hospital ventured to address their superior officers—otherwise the Committee of Management—they wish it to be known that such a step on their part would not have been taken had they not felt practically driven to it. The sister-in-charge had previously been informed of the grievance, but had seen fit to ignore it. Consequently, these "maidens in distress" informed their committee that unless a certain change was made in the institution they would themselves withdraw from the field of labour. On the other hand, it seems to have wounded the pride of the gentlemen in question that they should not have been requested to make an inquiry into this particular grievance, and certainly they did not appreciate the nurses definitely stating that either the committee of management must bring about the fulfilment of what they requested, or they themselves would make their exit. Suffice it to say that the nurses, while admitting their fault in their mode of address, still claim that their non-request for an inquiry lay in the fact that they did not wish the details in connection with the grievance to be exposed, and in their earnest endeavour to avoid this their error occurred. Ultimately these details were exposed, and, though the nurses gained the day, it cannot truly be said that mutual satisfaction exists. So acutely do they feel the unpleasant impression which these details (exposed, they consider, to an unnecessary extent) leave on the minds of all connected with it, that the majority of them, even though victorious, still intend leaving the institution.

### AFFLUENT BREADWINNERS.

"Another Who Loves Nursing" writes: I was glad to see "A Worker's" letter under the above heading in last week's issue, because it gives the opportunity for the discussion of an important subject, viz., whether nursing the poor should be allowed to become merely a "profession" instead of remaining a charity and a means of helping one's fellow creatures. Formerly the care of the sick and suffering was looked upon as a service rendered to One who said, "Inasmuch as ye did it unto one of the least of these My brethren, ye did it unto Me." Some of us are still old-fashioned enough to look upon it in this light, even though we may be sisters and nurses in large modern hospitals, and it is a real grief to see how the spirit of charity is being trampled out by the idea of our "being competitors in a struggle" to earn a living, by the race for promotion, by the petty jealousies, unkind gossip, frivolity, and slovenly work, which are directly due to forgetfulness or wilful ignoring of the fact that hospital nursing is not merely a means of earning a living but has a higher and holier side. Surely it is unnecessary to remind "A Worker" that a hospital is a charitable institution, founded primarily for the relief of the sick poor, and secondarily for the study of the prevention and cure of disease. They were never intended as a means of supporting "the moneyless members of the nursing profession." It would be as foolish to ask that a hospital should refuse paying probationers' fees, and should shut its doors to all candidates but those "who must of necessity do something to live," as it would be to propose such restrictions on the admission of men to the priesthood. Nurses to whom their salary is not necessary spend it chiefly on the patients in subscribing to convalescent homes, paying patients' fares, and, where the hospital is poor, in providing things for the wards. No doubt the pension fund is also a very good object, but the subscribers' money was given by them for the hospital and its patients, and if a nurse applies her superfluity of wealth to her hospital, she is certainly carrying out their intentions. The "dear friend" who is sneered at by "A Worker" because "she loves nursing," and does it "not because she is obliged to, but because she simply loves it," is the nurse I would have in my ward, and to whom I would trust my most exacting patients and worst cases when she has learnt her work rather than to one who merely regards nursing as a "profession" and herself as "a bread-winner." Nursing as a means of money-making is perfectly legitimate, but a nurse who becomes one merely for the sake of earning her living, or whose genuine love of the work is not her first object, is out of place in a hospital. She should take up private nursing, where the nurse and patient are in the relation of employed and employer, and where, to judge by the hundreds of advertisements for private nurses, the profession is not so overdone. I fail to see how "A Worker's" comparison of a society woman writing society paragraphs applies to nursing. No such writer pretends that it is her love of humanity or of literature which inspires her. The general public probably prefers to take its news from the "lady of wealth and position," because it wants facts as they are, and not works of imagination, on the simple principle that if one wanted a book on the manners and customs of a country one would choose one written by a man who had been there.

### The Book World for Women and Nurses.

[We invite Correspondence, Criticism, Enquiries, and Notes on Books likely to interest Women and Nurses. Address, Editor, THE HOSPITAL (Nurses' Book World), 28 & 29, Southampton Street, Strand, London, W.C.]

THE NURSING PROFESSION: HOW AND WHERE TO TRAIN. Edited by Sir HENRY BURDETT, K.C.B. (London: The Scientific Press.)

THIS helpful and much-needed book gives accurate and full information as to everything a nurse or nurse candidate may want to know. She may learn from its pages the kind of training employed in the various hospitals from Ballymena to Ballarat, or from Adenbrooke's to Johannesburg. Attractively bound and got up, teeming with every particular relating to the professional, industrial, and social side of nursing, there is no cut and dried dictionary flavour about it. Indeed, some portions of the book are very entertaining reading, and the chapters on nursing ethics might with advantage be read, marked, learned, and inwardly digested by the humble pro. and the qualified matron alike. The laws affecting nurses are set out in so simple a form as to be readily grasped by the most non-legal mind. The section devoted to customs as to contracts and broken engagements —of a strictly professional nature—should be conned by every nurse in private practice. Women's ethics on the law of contract are not always quite sound—and this particular chapter may be commended to feminine employers of nurses, as well as to the nurses themselves. Sir Henry Burdett has something, too, to say on the law of libel, and the personal responsibility of those who utter and repeat detrimental statements. His word in season on this point may save much trouble, since we of the profession know but too well how "sick room gossip" may translate itself into the slander case of the law court. No woman intending to train as a nurse, and no trained nurse, can afford to do without so useful a *vade mecum* as "The Nursing Profession," since it answers every problem as to where to train, how to train, and how to dispose of one's faculties after training. Intending nurse candidates, glancing through its pages, get a capital insight into their prospective work. A study of the various chapters opens out a clear and detailed vista of the life of a nurse. The seeker finds which hospital grants its pupils separate bed-rooms or cubicles, in which training school text books are provided for the staff, where sick cookery is taught, the exact number of hours off duty each day, week, and month, with full information as to special maternity and fever courses. The volume represents many months of careful painstaking research, and brings, in a nutshell, before the nurse all she can possibly want to know of her profession, and at so moderate a price that none can say, "I can't afford it." The editorial desks of papers devoted to feminine interests are flooded daily with letters whose vagueness supports the widespread assurance that the genus woman stands badly in need of a little business training. Inquiries such as these haunt the sub-editors of nursing papers: "Please give next week a list of hospitals which train nurses." The writer forgets that such a request would turn the next issue into a mere alphabetical list of institutions. Another asks, "I am going to India, please tell me what chances there are for nurses?" and so on *ad infinitum*. The hospital matron's desk is inundated with similar vague requests. Each year Guy's Hospital receives 3,000 applications from would-be nurses, and the London Hospital upwards of 2,000. The answering of this colossal correspondence—and the hundred and one questions included—constitutes an enormous amount of work, which would be reduced to a minimum were nurse candidates to provide themselves with this invaluable little handbook. Even so small a matter as the barring by two or three hospitals of probationers who fail to reach the imposing height of five feet three inches is set forth in accurate detail. Time, postage, and waiting will be saved by a glance through the sections devoted to Continental institutions employing trained nurses, and by the explicit information given as to the leading training schools of Australasia, the United States, and South Africa. Full particulars are set forth of the army and navy nursing services, employment for nurses in India, added to all the openings in hospitals and institutions of the provinces, and of Ireland and Scotland. It is quite safe—without incurring any of the risks which commonly fall to the lot of the prophet—to predict that of all the books interesting to nurses, which have been or will be published during the year, none will fill so useful an every-day purpose as guide, philosopher, and friend as will this attractive scarlet-bound book, "The Nursing Profession."

A PRIMER OF PSYCHOLOGY AND MENTAL DISEASE FOR USE IN TRAINING SCHOOLS FOR ATTENDANTS AND NURSES AND IN MEDICAL CLASSES. By C. B. BURR, M.D. Second Edition. Pp. ix.—116. (Sampson Low, Marston, and Co. Price $1.)

IN his preface the author states that "the association of the concept embarrassment and the concept commiseration has produced the judgment to write this unambitious little book." Whether the average nurse or attendant in our county asylums would quite grasp the meaning of this, it is certain that in 1893, the date of the first edition of Dr. Burr's book, manuals for the use of students attending mental nursing classes were few and far between, perhaps non existent; but in 1897 this charge could not have been made, as several treatises were in the field, notably Dr. Harding's admirable little work, which is, however, intended for nurses only. It can easily be understood that Dr. Burr's pupils hailed the appearance of the book with satisfaction, for it is well got up and pleasantly written, and conveys a large amount of information in its 88 pages of letterpress proper. On this side of the Atlantic it might be said that the work is deficient in its purely "nursing" aspect, and that on the other hand it contains much which a nurse cannot reasonably be expected to know, and which she might have some difficulty in following. For instance, it is stated that in paranoia "memory is unimpaired, organic memory and personalty changed, ideation unimpaired, and no incoherence in grouping of concepts." But there are always in every class a few nurses who thirst for knowledge, and who are anxious to go further than their nursing lectures lead them. To these this little primer will be extremely useful and may be warmly recommended. On page 37 it is stated that in mania there is no tendency to suicide. Rare the tendency may be; but we feel sure that Dr. Burr's statement is of much too sweeping a nature, and that patients who cannot be classed elsewhere than under the "mania" group are occasionally suicidal. As to the secondary object of the work—namely, as a guide to medical students—it may be said that it is insufficient for those of our undergraduates who intend presenting themselves at the higher examinations; but that it would, with some samplification, be a most useful book for those who are satisfied with our minor qualifications. For this purpose the section on epilepsy should be expanded, and a new one on moral insanity added, and both of these should be carefully viewed from their medico-legal aspect, a point in which our medical witnesses (not experts) are often extremely deficient. Further, it would be essential to add a chapter on the purely medical treatment of the varieties of insanity.

### Travel Notes.

By Our Travelling Correspondent.

### X.—THE CARNIVAL.

Before quitting Nice and its neighbourhood for a time, which we must soon do, I must tell you a little about the Carnival, which still survives in Nice, though in Rome it is but a sorry affair. Why this should be it is difficult to say. In Rome and the other big cities of North Italy King Carnival's reign is nearly at an end; he drags out a precarious and meagre existence, and ere long he will certainly abdicate. In Nice he still reigns with proper grandeur and majesty, and it is there, and there only, that Carnival can be seen at all adequately in these degenerate days. The price of apartments and hotels runs up so enormously at this season that one holds one's breath, and feels a certainty of one's ultimate destination being the workhouse!

### The Battle of Flowers.

The ball opens with the battle of flowers, when all the fashionable world drives on the Promenade. I must not stay to tell you of the splendid floral decorations, but must leave it to your imagination. The offi cers of the garrison are generally very magnificently built up. Last time I saw them they were in a species of huge brake, or perhaps it was a wagon, embowered in masses of violets and branching mimosa; even the spokes of the wheels were hidden in violets, and all the trappings of the horses were similarly decorated. The warriors themselves were most active in warfare. Having huge baskets of small bunches of flowers as ammunition, they darted from side to side and pelted everybody with military precision. It is the etiquette of Carnival to return your enemy's fire; indeed if this were not done action must cease through the expenditure of ammunition. It is a very gay and pretty scene, but only suited to a sunny land. When I saw the Eastbourne Battle of Flowers I felt the entire thing to be a mistake; we have not the sun, the brilliant surroundings, the plentiful flowers, and, above all, the genius of the English people is not suited to graceful fooling. We take our pleasures sadly, as our foreign neighbours say, and we cannot alter our national character. The bicycle races, with all the machines festively wreathed and trimmed, did not please me much. There is something so loudly and offensively modern about a bicycle that it does not assimilate with the ancient court of King Carnival.

### Confetti Throwing.

Before the battle of the confetti you must provide yourself with a mask and domino; it is impossible to pass through the streets without a mask, or you would be blinded. Words fail me to describe the staring horror of these wire masks; the ladies' are painted into a ghastly appearance of a face, the eyes of which stare stonily at the passers by in a manner hideous to behold. I have sketched one or two for you to see; the gentlemen's are not quite so ugly because they are left in naked simplicity; even through this wire gauze the chalk from the confetti penetrates, and one's eyes and nose stream. If your invalid would like to see the fun, and is equal to it, the best plan is to take seats on the stands in the Place Massena; choose them high up so as to be a little free from the attention of the confetti throwers, but if you are strong and not nervous the amusing thing is to go down into the thick of the fray, to throw and be thrown at, and enter into the full spirit of the whole thing. The maskers are wild with revelry, but it is a revelry that never passes the bounds of decorum; roughness or rudeness is unknown. At first one feels a little shy, but gradually realising the perfection of one's disguise, the feeling wears off. On no account show offence at any thing; "at Rome do as the Romans do" must be the motto for the day. A gay masker will perhaps come along and put his arm round your waist saying either in French or Italian "By your, leave lovely girl," and will waltz some 10 or 12 yards with you to the sound of a band which is playing near. The "lovely girl" may be some staid and sober matron of 60, but this is a detail; no feature is exposed to confess the ravages of time.

### The Procession.

The procession is extraordinary, some 50 or 60 enormous cars, led by King Carnival, his sturdy consort, and a hopeful family of young carnivals. The occupants of the cars have great advantages in confetti throwing, and the only objects of their mercy are the soldiers, who are not allowed to mask,

Masks at The Carnival.

so that the friendly, gay, southern nature holds a truce with them, for a shovelful of chalky confetti in unmasked eyes might very possibly end in total blindness. The warfare begins and ends with the firing of guns from the fort and at the conclusion one is very thankful to go home to tea and rest preparatory to the evening festivities, which consist of illuminations, fireworks, and the destruction of King Carnival by a fiery death in the old market place.

### The Illuminations and Bonfire.

The decorations are really lovely, but especially in the old quarter, where the bonfire takes place; thousands of Chinese lanterns strung on wreaths cross and recross the old square and adjacent streets; the crowd is enormous but very well conducted. I speedily ensconced myself for safety against a wall; the gentleman I was with had his wife, and it is quite impossible to protect two women, so I cut myself adrift and came to no harm. The only danger is when a large group of maskers suddenly start on a wild dance, because their numbers gradually augment and the pressure from behind prevents their stopping. That occurred once to me, but a sergeant and three privates were near to me, and they linked arms, putting me in the middle, and though the pressure was fearful, and I felt as if my last hour was come, they stood firm and I emerged safe and sound. In thanking them I unmasked for a moment, as that is considered an act of courtesy. At ten o'clock his majesty expires; he is padded with straw, tar, and squibs, and makes a noble blaze. Fireworks are let off, everyone screams and shrieks, and Carnival is over. It is a sight well worth seeing, and I counsel you to miss nothing of the fun if possible.

HINTS FOR CONTINENTAL TRAVELLERS.

Always make sure that your *femme du ménage* does not clear out the wood ashes from your stoves. Insist upon these being left. It is a common practice to clear them away and sell them to the laundresses. A fire does not really burn well until you have the accumulation of two or three weeks, this becomes heated throughout and, smouldering slowly, only requires a few puffs from the bellows to blaze up, and retaining the heat is a great economy.

RAILWAY TIPS.

You must always remember that there are not innumerable porters at the stations as in England, who are bound to attend to you. It is far otherwise, which makes them independent. I find a judicious expenditure of 50 c. pieces insures me plenty of attention, and after all one is not greatly the poorer at the end of the journey, and one has effected a wonderful saving in time and temper.

### TRAVEL NOTES AND QUERIES.

RULES IN REGARD TO CORRESPONDENCE FOR THIS SECTION.—All questioners must use a pseudonym for publication, but the communication must also bear the writer's own name and address as well, which will be regarded as confidential. All such communications to be addressed "Travel Editor, 'Nursing Mirror,' 28, Southampton Street, Strand." No charge will be made for inserting and answering questions in the inquiry column, and all will be answered in rotation as space permits. If an answer by letter is required, a stamped and addressed envelope must be enclosed, together with 2s. 6d., which fee will be devoted to the objects of the "Hospital Convalescent Fund." Any inquiries reaching the office after Monday cannot be answered in "The Mirror" of the current week.

DAVOS (Rubrio).—First-class single by shortest route £6 13s. 9d.; 2nd, £4 12s. 3d. It is not an expensive place considering its popularity. There is a large choice of hotels. I know best the Schweizerhof; very reasonable pension terms. Dr. Lunn recommends the Pension Stula, still cheaper.

ROME (Daisy).—The book you mean is "Walks in Rome," by Augustus Hare, price 21s., published by Daldy, Isbister, and Co., 56, Ludgate Hill, E.C.

VERONA (Victoria Cross).—I think you might safely reside in Verona if all you tell me is correct. Fortunately, economy is not imperatively necessary. Therefore choose rooms with a full south aspect, and high up is always desirable. Take plenty of warm curtains, and especially English blankets (these can go out by Petite Vitesse). Do not stint for fires, and I think you will find it comfortable and beneficial.

SPAIN (Flauto Magico).—Travelling there is still open to some objections, and I certainly should not recommend it to any but the robust. Malaga, however, is a delightful health resort for those suffering from chest trouble, and being much frequented by the English it begins to be really comfortable. North Spain is extremely cold and not to be thought of.

PARIS (Soleil).—You are right that Paris has much more sun than London, but you must not imagine it to be much warmer. It can be frightfully cold anywhere but in well appointed rooms and with unlimited fires.

VENTNOR (Suburban).—I never think any English seaside place equal in value to those on the Continent, because of our lamentable lack of sun, and lodgings are very dear. Why not try Vevey, in Switzerland, near to Montreux. If you do not mind the journey it would be quite the place for you. Good pensions may be had from 6 francs.

ROME (Perplexed).—The water is excellent, and with ordinary precautions there is no fear of Roman fever. It is because non-inhabitants are so obstinate and wilful that they sometimes suffer before they become acclimatised. One of the predisposing causes is over-fatigue, engendered by perpetual sight-seeing.

LOURDES (Evening Star).—I am not aware of any curative properties in the spring at Lourdes other than those attributed to its miraculous healing powers. An article will shortly appear in the "Mirror" describing this wonderful modern shrine. Enormous pilgrimages, among the saddest sights in the world, are organised from all parts of the world, many times every year, when suffering in all its worst phases may then be exhaustively studied.

*For Travel Advertisements see page* xviii.

### Notes and Queries.

**The contents of the Editor's Letter-box have now reached such unwieldy proportions that it has become necessary to establish a hard and fast rule regarding Answers to Correspondents. In future, all questions requiring replies will continue to be answered in this column without any fee. If an answer is required by letter, a fee of half-a-crown must be enclosed with the note containing the enquiry. We are always pleased to help our numerous correspondents to the fullest extent, and we can trust them to sympathise in the overwhelming amount of writing which makes the new rules a necessity.**

**Every communication must be accompanied by the writer's name and address, otherwise it will receive no attention.**

*Nursing in Paris.*

(197) Will you kindly tell me (1) How to obtain rules of the British Hospital in Paris; (2) If there is any other hospital or home there for English certificated nurses; (3) What the salaries are—whether better than in England; (4) Is it necessary to speak French fluently or not? —*Nellie.*

1. Apply the Matron, the Hertford Hospital, Rue de Villiers, Levallois-Perret, Paris. 2. The Secretary, Y.W.C.A., 88, Faubourg St. Honoré, Paris, would advise about board and residence. 3. The salaries are less and the position inferior to those of nurses in England. (4) No; though of course it is an advantage to be conversant with the language.

*Maternity Training.*

(198) Can you tell me where in Manchester I could have coaching lessons for the L.O.S. examination?—*Street.*

We cannot give you the names of any private "coaches" for the L.O.S. in Manchester, but you may be trained for it at any good maternity nursing school. Apply to the matrons of the maternity hospitals for particulars. The address of the Manchester Maternity Hospital is 60, Upper Brook Street, Manchester, and that of St. Mary's Hospital is Quay Street.

*Enemata.*

(199) There appeared in the medical section of your paper a small article describing a result (high temperature, rash, &c.) sometimes occurring after giving a copious purgative enema; could you let me know which week it was in that I might try and procure a number?—*E. C. MacG.*

Dr. Oliver's article "On toxic materials released from the fæces by enemata and poisoning the system," appeared in THE HOSPITAL on September 3rd, 1898.

*A Year's Training.*

(200) Is there any hospital or infirmary of 50 beds where I could get a year's general training? My age being 37 I am over the age limit of most places. I have had two years' training at Plaistow Nurses' Home. I can pay a small premium.—*Nurse Ellen.*

Inquiry might be made at one or other of the following:—The Cheltenham General Hospital (beds 82), special probationers received for one year; premium, £15 15s. Bedford General Infirmary, 60 beds; paying probationers received; age over 22. Bristol Hospital for Sick Women and Children, beds 102; paying probationers received without limitation of age. West Kent General Hospital, Maidstone, beds 54. One year probationers pay a premium of £33. Age limit 40.

*Ward Floors.*

(201) Would you kindly tell me what can be done to ward floors which have been stained and varnished? The varnish has nearly all worn off, and the floors, instead of looking polished after beeswax and turpentine has been applied, are only dull and black.—*Inquirer.*

Scrub all oily polish off with strong soda and water. Then either restain and varnish or repolish with the beeswax and turpentine. Elbow grease is an equally important factor in securing a good polish.

*Fever Certificate.*

(202) Would you kindly tell me if at the City of Glasgow Fever and Small-pox Hospitals, Belvedere, a three years' certificate may be obtained, or if only a fever training is given?—*A Reader of* THE HOSPITAL.

At the end of a probationer's second year, if she passes a satisfactory examination, she receives "A Certificate of Proficiency in Fever Nursing." This is the only certificate given at this hospital.

*Ear Drums.*

(203) Can you kindly tell me if Wilson's Common Sense Ear Drums, as advertised in the monthly magazines, if used, would give any real good in a case of perforation of the ear?—*Nurse.*

Consult an aural surgeon, and don't waste money trying mechanical contrivances until prescribed by a competent authority.

*Superannuation Act.*

(204) Could you kindly let me know what the Poor Law Officers' Superannuation Act and Amendment Act are, and in what way do they affect nurses? Is it possible for nurses who have had workhouse infirmary training to obtain posts in hospitals afterwards, that is providing they possess a certificate?—*E. E.*

You may obtain a copy of the "Superannuation Act" at any law stationer's for 1d. This Act regulates the allowances deducted from the salaries of nurses and other Poor Law officials in order to provide a fund for granting superannuation allowances. (2) Some of the workhouse infirmary certificates rank with those of first-rate hospitals, and would qualify their holders for the best posts.

*Bury St. Edmunds.*

(205) Could you inform me (1) what are the rules and hours for a probationer at the Bury St. Edmunds Hospital? also (2) if they find uniform?—*N. A.*

1. Write to the Matron for particulars: or refer to "The Nursing Profession: Where and How to Train" (The Scientific Press, price 2s.). 2. The indoor uniform is provided; the outdoor is not. You should have sent address as well as name.

*Hypodermic Syringe.*

If Sister Gertrude will send name and address, in compliance with our rules, her question will be answered.

## Figures and Tables

**Figure f1:**
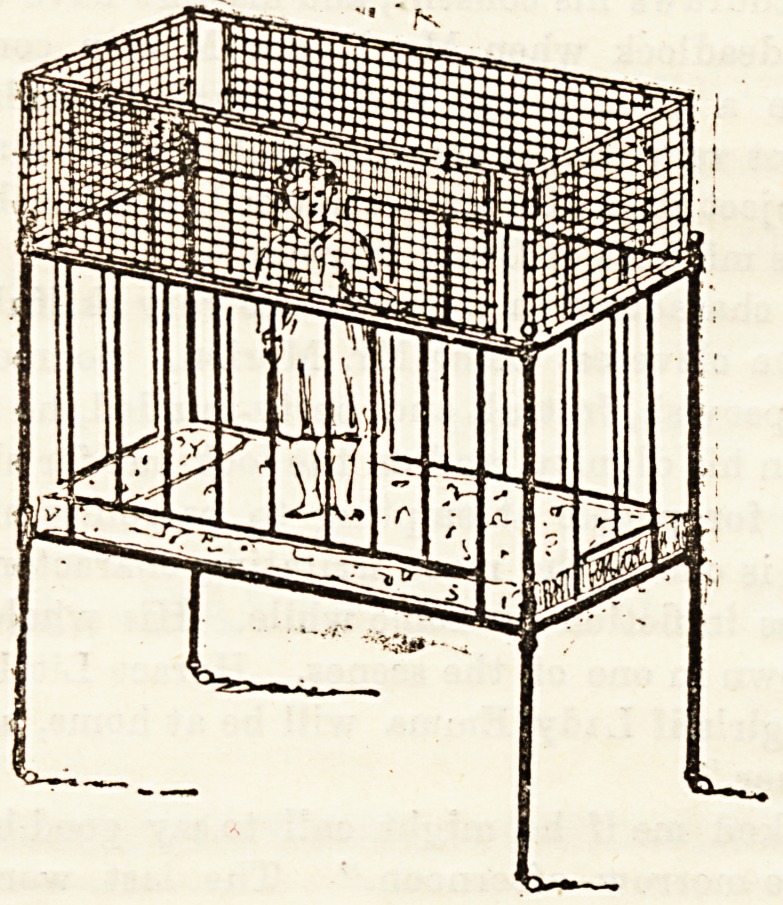


**Figure f2:**
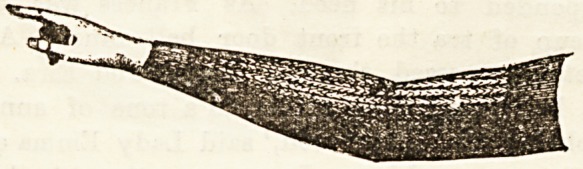


**Figure f3:**